# Real-World Study Assessing Sensitivity and Clinical Utility of a Host-Protein Test in Adult Emergency Department Patients With Blood Culture Ordered

**DOI:** 10.1016/j.acepjo.2025.100255

**Published:** 2025-09-30

**Authors:** Alexandra LoVerde, Monica Ghitan, Hannah Bodenstein, Manan Christian, Lior Kellerman, Boris Lebedenko, Eran Eden, Tanya Gottlieb, Sergey Motov

**Affiliations:** 1 Department of Emergency Medicine, Maimonides Medical Center, Brooklyn, New York, USA; 2 Division of Infectious Disease, Maimonides Medical Center, Brooklyn, New York, USA; 3 Division of Information Services, Maimonides Medical Center, Brooklyn, New York, USA; 4 Department of Pathology, Maimonides Medical Center, Brooklyn, New York, USA; 5 MeMed, Haifa, Israel; 6 MeMed US, Andover, Massachusetts, USA

**Keywords:** host-response test, bacterial vs viral, antibiotic stewardship, clinical utility, real-world study

## Abstract

**Objectives:**

Difficulty in distinguishing bacterial from viral infections leads to suboptimal patient management. MeMed BV (MMBV) is a Food and Drug Administration-cleared host-protein test with >98% negative predictive value (NPV) for distinguishing bacterial (including coinfection) from viral infections. This study aimed to validate MMBV’s sensitivity compared with blood cultures and assess clinical impact over time.

**Methods:**

At Maimonides Medical Center emergency department (ED) (March 2022 to March 2024), adults were included if both MMBV and blood cultures were ordered at the physician’s discretion. Sensitivity was evaluated against clinically relevant blood cultures. Alignment was defined as not prescribing antibiotics for MMBV viral results (score <35) and prescribing for bacterial (score >65). Outcomes included prescribing alignment, antibiotic prescription, hospital admission, and 7-day ED-return visits. Outcomes were compared across matching time periods: April 2022 to March 2023 (period 1) and April 2023 to March 2024 (period 2).

**Results:**

The study included 681 adults (median age, 72 years; 52.9% male), with 58 clinically relevant blood cultures, 29 contaminated cultures, and 594 negative cultures. MMBV attained 96.4% sensitivity (95% CI, 87.0-99.7) and 98.6% NPV (94.5-99.9). Comparing period 1 (n = 353) with period 2 (n = 291), antibiotic prescription among patients with viral MMBV results decreased from 80.7% to 61.4% (*P* = .017), hospital admissions declined from 77.3% to 61.4% (*P* = .056), and there was no significant change in return visits. Antibiotic prescription to patients with bacterial MMBV results increased from 93.0% to 98.1% (*P* = .012) without impacting admission or return visits.

**Conclusion:**

MMBV demonstrated high sensitivity. Increased test alignment is associated with reduced antibiotic prescription and hospital admission in viral MMBV cases without compromising safety.

**The Bottom Line**

Difficulty in determining infection etiology leads to suboptimal patient management. MeMed BV (MMBV) is a Food and Drug Administration-cleared host-protein test for distinguishing bacterial from viral infections. Here, we validated MMBV’s performance compared with clinically relevant blood cultures and assessed its clinical impact. At Maimonides Medical emergency department (2022-2024), there were 681 adults for whom MMBV and blood cultures were ordered at the physician’s discretion. MMBV demonstrated 98.6% negative predictive value (95% CI, 94.5-99.9). There was a significant decrease in antibiotic prescription and admissions for patients with viral MMBV results across time. MMBV demonstrated high performance associated with improved patient outcomes.

## 1 Introduction

### 1.1 Background

Distinguishing between bacterial and viral infections remains a significant challenge in emergency department (ED) settings. Diagnostic uncertainty leads to unnecessary antibiotic use, contributing to antimicrobial resistance, avoidable drug reactions, and health care costs. Conversely, failure to promptly recognize bacterial infections leads to delayed antibiotic treatment, with avoidable complications and prolonged hospital stays.

### 1.2 Importance

The current gold standard for detecting bacteremia is blood culture. Despite technological improvements and emerging data on best practices, blood cultures have limitations, including prolonged time to results (24-48 hours), low sensitivity leading to false negatives, and contamination resulting in false positives.^1^, ^2^, ^3^ Especially when resources are limited,^4^ accurately stratifying the patients for whom blood cultures should be ordered can minimize unneeded testing and reduce the burden of managing the consequences of contaminated samples. There is a pressing need for rapid and reliable tools to help differentiate between bacterial and viral infections and guide timely, optimized diagnostic workup and antibiotic treatment decisions in the ED.^5^

MMBV is a host-protein test that integrates circulating levels of tumor necrosis factor-related apoptosis-inducing ligand (TRAIL), interferon gamma-induced protein-10, and C-reactive protein (CRP) to generate a bacterial likelihood score. MMBV was extensively validated against a clinical adjudication-based reference standard, demonstrating sensitivity and specificity >90%, and a negative predictive value (NPV) >98%.^6^, ^7^, ^8^ Cumulating clinical utility studies indicate that MMBV optimizes antibiotic treatment decisions, with the net impact on prescription rates dependent on baseline practice. Importantly, reduced prescription to patients with viral MMBV scores is not associated with more ED-return visits, supporting safe antibiotic stewardship.^9^^,^^10^ In parallel, increased prescription to patients with bacterial MMBV scores is associated with fewer ED-return visits, indicating improved patient outcomes.^9^^,^^10^

### 1.3 Goal of this investigation

Previously, we reported on the operational introduction of MMBV into our busy ED that manages high-risk populations.^11^ Here, we validate MMBV’s sensitivity compared with blood cultures and assess its clinical impact over time.

## 2 Methods

### 2.1 Study design

This pragmatic retrospective study was designed to evaluate MMBV’s diagnostic accuracy and its clinical impact over time. ED patient records were reviewed from March 22, 2022 to March 31, 2024 and analyzed across (1) the entire period and (2) 2 matching 12-month time periods: April 1, 2022 to March 31, 2023 (period 1) and April 1, 2023 to March 31, 2024 (period 2).

### 2.2 Setting and selection of participants

The study was conducted at Maimonides Medical Center, an academic hospital with approximately 120,000 annual ED visits, and the largest hospital in Brooklyn, New York, USA. The study was approved by the institutional review board (MMC IRB 2024-06-10).

Adult patients aged ≥18 years were included, for whom both MMBV and blood culture testing were ordered. This population is expected to encompass patients for whom there is a need to rule out bacterial infection.

Patients for whom blood cultures were not processed by the laboratory were excluded. Reasons for not processing a blood culture order included insufficient sample volume, documentation errors, and physician cancellation. MMBV results were not available at the time the physician cancelled.

### 2.3 Exposures

#### 2.3.1 MMBV

**Figure 1:**
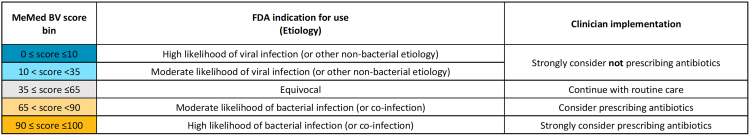
MeMed BV test results and interpretation.

MMBV generates a bacterial likelihood score ranging from 0 to 100, with higher scores indicative of bacterial infection, including bacterial-viral coinfection (Fig 1). The test was performed using serum samples run on the MeMed Key platform.

In-training service of ED and lab staff regarding MMBV took place in March 2022, together with establishing an order set and an interface with the lab. Physicians were trained via a 3-part lecture series that included an introduction to the test, supporting evidence, and practical application in the ED (ie, ordering procedure, interpretation of the results (Fig 1), and documentation). MMBV was ordered at the physician’s discretion.

#### 2.3.2 Clinical adjudication of blood cultures

For every case with a microbiological finding in a blood culture, an infectious disease physician (M.G.) with 20+ years of experience reviewed the medical record (clinical and laboratory data) and adjudicated if the growth was clinically relevant or a contamination. The adjudicator was blinded to MMBV results.

### 2.4 Outcomes

The primary study outcome was MMBV’s diagnostic accuracy, with clinically relevant positive blood culture results serving as the reference standard for bacterial infection.

Secondary outcomes included the following:

• Alignment of ED antibiotic prescription with MMBV test results, defined as not prescribing antibiotics to patients with a viral MMBV score (<35) and prescribing antibiotics to those with a bacterial MMBV score (>65). For patients with equivocal MMBV results (35-65), alignment cannot be defined, as this result is not actionable. Therefore, these cases are not included in the alignment analyses.
• Antibiotic prescription rates in the ED.
• Hospital admission rates.
• Mean length of stay in the hospital (days).
• Return ED visits within 7 days.

### 2.5 Data analysis

Clinical and demographic data were summarized as descriptive statistics. Continuous variables were reported as median and interquartile range and compared using the Mann-Whitney U test. Categoric variables were expressed as proportions, with differences assessed using Fisher exact tests. A *P* value < .05 was considered statistically significant. Statistical analysis was performed using Python version 3.9.19.

Sensitivity, specificity, NPV, and positive predictive value were calculated to assess diagnostic accuracy. The statistical framework is described in the Supplementary Materials. The diagnostic accuracy analysis and findings are reported in alignment with Standards for Reporting Diagnostic Accuracy guidelines; the checklist is provided in Supplementary Materials.

## 3 Results

### 3.1 Study population

**Figure 2:**
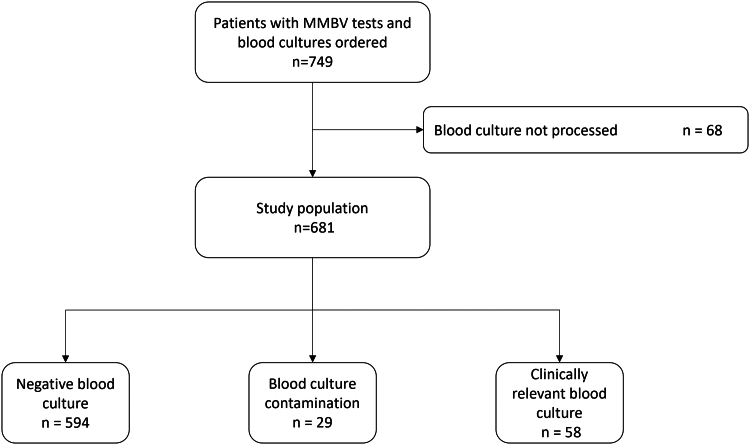
Blood culture results. There were 68 blood cultures ordered that were not processed. Clinical relevance of the positive blood culture was adjudicated by M.G. based on all abstracted clinical and laboratory data.

The study included 681 adult patients with blood cultures processed (Fig 2). The median age was 72 years (Q1-Q3, 56-83), 52.9% were male (Table). Chief complaints were fever (24.4%), dyspnea (19.4%), and altered mental status (10.4%). Most patients (86.0%) were admitted to the hospital, with a mean stay of 8.8 days (SD, 10.5). Antibiotics were frequently prescribed at the ED (89.9%). The most common discharge diagnosis was infectious disease (33.6%).

**Table:** Patient characteristics in the study population stratified by MeMed BV results.

		All (N = 681)	Bacterial (N = 461)	Equivocal (N = 82)	Viral (N = 138)
Demographics	Age (y), median (IQR)	72.0 (56.0, 83.0)	72.0 (58.0, 84.0)	73.0 (57.0, 84.8)	69.0 (46.0, 80.0)
	Sex (male), n (%)	360 (52.9%)	251 (54.4%)	34 (41.5%)	75 (54.3%)
Current illness	Temperature (C), mean (SD)	37.6 (1.1)	37.6 (1.1)	37.2 (0.9)	37.6 (1.2)
	ED LOS (h), mean (SD)	8.4 (6.0)	8.5 (6.0)	9.0 (7.3)	7.9 (5.0)
Chief complaint	Fever, n (%)	166 (24.4%)	122 (26.5%)	13 (15.9%)	31 (22.6%)
	Dyspnea, n (%)	132 (19.4%)	81 (17.6%)	24 (29.3%)	27 (19.7%)
	Altered mental status, n (%)	71 (10.4%)	49 (10.6%)	12 (14.6%)	10 (7.3%)
	Weakness/fatigue/lethargy, n (%)	48 (7.1%)	35 (7.6%)	6 (7.3%)	7 (5.1%)
	Cough, n (%)	33 (4.9%)	16 (3.5%)	3 (3.7%)	14 (10.2%)
	Abdominal pain, n (%)	31 (4.6%)	24 (5.2%)	2 (2.4%)	5 (3.6%)
Tests	CBC ordered, n (%)	674 (99.0%)	456 (98.9%)	81 (98.8%)	137 (99.3%)
	WBC, median (IQR)	10.3 (7.2, 14.5)	11.6 (8.4, 15.8)	9.3 (6.5, 12.4)	7.1 (5.5, 9.4)
	Procalcitonin, median (IQR)	1.1 (0.5, 4.2)	1.2 (0.6, 4.6)	0.6 (0.4, 1.1)	0.9 (0.4, 2.1)
	CRP ordered, n (%)	83 (12.2%)	47 (10.2%)	11 (13.4%)	25 (18.1%)
	CRP (mg/dL), median (IQR)	5.1 (1.7, 13.4)	11.4 (4.7, 15.7)	4.7 (1.3, 10.8)	1.3 (0.6, 3.0)
	Broader viral PCR test ordered, n (%)	64 (9.4%)	47 (10.2%)	9 (11.0%)	8 (5.8%)
	Rapid viral panel test ordered, n (%)	389 (57.1%)	270 (58.6%)	41 (50.0%)	78 (56.5%)
	Influenza A, n (%)	15 (3.9%)	5 (1.9%)	0 (0.0%)	10 (12.8%)
	Influenza B, n (%)	2 (0.5%)	1 (0.4%)	0 (0.0%)	1 (1.3%)
	COVID-19, n (%)	36 (9.3%)	14 (5.2%)	4 (9.8%)	18 (23.4%)
	RSV, n (%)	5 (1.3%)	3 (1.1%)	1 (2.4%)	1 (1.3%)
Microbiological testing	Blood culture ordered, n (%)	681 (100.0%)	461 (100.0%)	82 (100.0%)	138 (100.0%)
	Blood culture results: contaminant, n (%)	29 (4.3%)	22 (4.8%)	1 (1.2%)	6 (4.3%)
	Blood culture results: negative, n (%)	594 (87.2%)	386 (83.7%)	78 (95.1%)	130 (94.2%)
	Blood culture results: positive, n (%)	58 (8.5%)	53 (11.5%)	3 (3.7%)	2 (1.4%)
MMBV	MMBV score, median (IQR)	90.0 (47.0, 99.0)	97.0 (89.0, 100.0)	49.0 (42.0, 57.0)	6.0 (2.0, 17.0)
	MMBV result: bacterial, n (%)	461 (67.7%)	461 (100.0%)	0 (0.0%)	0 (0.0%)
	MMBV result: equivocal, n (%)	82 (12.0%)	0 (0.0%)	82 (100.0%)	0 (0.0%)
	MMBV result: viral, n (%)	138 (20.3%)	0 (0.0%)	0 (0.0%)	138 (100.0%)
	Alignment, n (%)	477 (79.6%)	441 (95.7%)	NaN	36 (26.1%)
Hospitalization	Hospital admission, n (%)	586 (86.0%)	421 (91.3%)	66 (80.5%)	99 (71.7%)
	Hospitalization duration, mean (SD)	8.8 (10.5)	9.4 (11.6)	8.3 (6.7)	6.5 (6.3)
Antibiotics	Antibiotics prescribed in ED, n (%)	612 (89.9%)	441 (95.7%)	69 (84.1%)	102 (73.9%)
Discharge diagnosis	Infectious diseases, n (%)	229 (33.6%)	170 (36.9%)	19 (23.2%)	40 (29.0%)
	Respiratory disorders, n (%)	67 (9.8%)	36 (7.8%)	14 (17.1%)	17 (12.3%)
	Cardiovascular disorders, n (%)	57 (8.4%)	34 (7.4%)	8 (9.8%)	15 (10.9%)
	Hematological and immunologic disorders, n (%)	56 (8.2%)	44 (9.5%)	5 (6.1%)	7 (5.1%)
	Endocrine and metabolic disorders, n (%)	49 (7.2%)	35 (7.6%)	7 (8.5%)	7 (5.1%)
Return visits	Return visit within 7 d, n (%)	54 (7.9%)	37 (8.0%)	9 (11.0%)	8 (5.8%)

CBC, complete blood count; CRP, C-reactive protein; ED, emergency department; LOS, length of stay; MMBV, MeMed BV; NaN, not a number; WBC, white blood cell count.

Regarding laboratory diagnostic tests other than blood cultures, the most ordered were complete blood count (99.0%), CRP (12.2%), rapid viral PCR tests (57.1%), and broader viral PCR tests (9.4%). Respiratory virus detection was low among the rapid viral PCR tests, with 3.9% testing positive for influenza A, 0.5% for influenza B, 9.3% for COVID-19, and 1.3% for RSV.

### 3.2 Sensitivity of MMBV in comparison to clinically relevant blood cultures

Blood cultures were adjudicated as clinically relevant in 58 (8.5%) cases, negative in 594 (87.2%), and classified as contaminants in 29 (4.3%) cases. Compared with clinically relevant blood cultures, MMBV performed with a sensitivity of 96.4% (95% CI, 87.0-99.7) and a NPV of 98.6% (94.5-99.9) (Table S1). Two of the 58 patients with a clinically relevant blood culture received a viral MMBV result, and 3 received equivocal MMBV results (Table S2).

### 3.3 Association between MMBV results and clinical outcomes

Higher antibiotic prescription rates (95.7% vs 73.9%; *P* < .001), higher hospital admission rate (91.3% vs 71.7%; *P* < .001), and longer mean length of hospital stay (9.4 vs 6.5; *P* = .016) were associated with bacterial MMBV compared with viral MMBV results (Table).

To assess if there was increasing alignment between MMBV result and antibiotic prescribing across time, we divided the study population into 2 cohorts from matching 12-month periods: April 2022 to March 2023 (period 1; n = 353) and April 2023 to March 2024 (period 2; n = 291). Demographics and distribution of chief complaints were similar between the 2 time periods (Table S3). Rates of other tests ordered, other than rapid viral PCR, were also comparable across time periods. Although there were significant increases in rapid viral PCR ordering, there was not a significant increase in viral detection (*P* > .108). There was an increase in alignment between MMBV viral results and not prescribing antibiotics in period 2 (19.3% vs 38.6%; *P* = .017). Alignment between MMBV bacterial results and prescribing antibiotics was high in both periods, with a significant increase in period 2 (93.0% vs 98.1%; *P* = .012).

**Figure 3:**
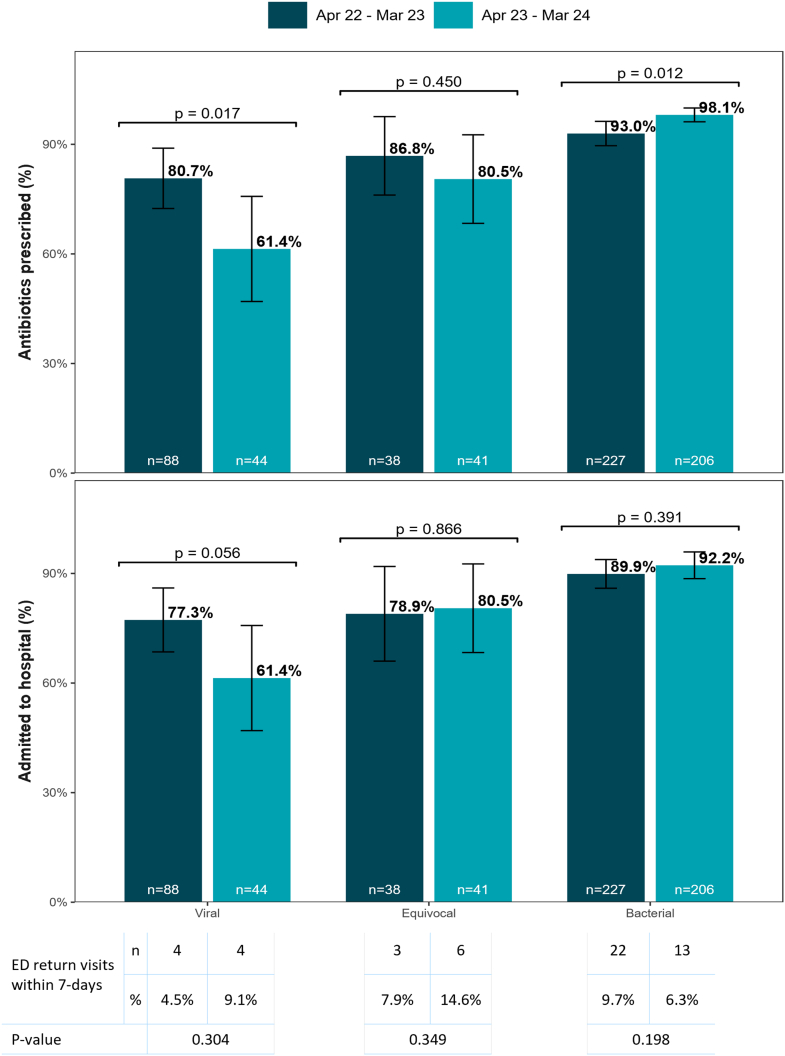
Comparison of clinical outcomes across 2 time periods stratified by MeMed BV result.

In the viral MMBV cases, alongside the aforementioned increase in prescribing alignment, not only antibiotic prescription (80.7% vs 61.4%; *P* = .017) but also hospital admission rates (77.3% vs 61.4%; *P* = .056) decreased in period 2 (Fig 3). There was not a significant change in ED-return visits within 7 days across the 2 time periods (4.5% vs 9.1%; *P* = .304).

In the bacterial MMBV cases, there was a significant increase in the rate of antibiotic prescription (93.0% vs 98.1%; *P* = .012), without significant changes in hospital admission (89.9% vs 92.2%; *P* = .391) or ED-return visits within 7 days (9.7% vs 6.3%; *P* = .198) across the time periods.

## 4 Limitations

First, test ordering was at the physician’s discretion, potentially introducing a selection bias toward early adopters and an information bias (as not all data variables are available), an intrinsic feature of the pragmatic nature of this study. Second, this was a single-center study conducted in a high-acuity ED that manages a complex patient population. Clinical practices and patient characteristics may differ from those of other institutions. Additional studies are required in other centers to establish the generalizability of the findings. Finally, it is possible that unmeasured confounding factors, such as antibiotic stewardship initiatives and institutional policy changes, may have contributed to the observed changes in clinical outcomes across the 2 time periods. However, it is notable that almost all measured variables were comparable across the 2 cohorts.

## 5 Discussion

In this pragmatic real-world study, we evaluated the diagnostic accuracy of MMBV in adults for whom blood cultures were ordered—a population with suspected infection for whom there is a concern for bacterial infection. MMBV achieved high diagnostic performance when compared against clinically relevant blood cultures, with an NPV of 98.6%. In addition, we showed that over time, test availability was associated with increased alignment between MMBV result and antibiotic prescribing and, importantly, with improved clinical outcomes.

The sensitivity observed here (96.4%) aligns with the >90% reported in multiple diagnostic accuracy studies that compared MMBV results with a clinically adjudicated reference standard.^6^, ^7^, ^8^ Of note, we do not highlight specificity, as it is well known that many patients with bacterial infections do not yield a clinically relevant blood culture.^7^^,^^12^ MMBV’s high performance against clinically relevant cultures raises the possibility that the test could help stratify which patients merit culture testing, particularly when culturing resources are limited.

In this study, 88% of the patients yielded actionable MMBV results, namely bacterial (including coinfection) or viral (nonbacterial). Among the 12% of patients with equivocal scores, there were 3 cases that had a clinically relevant blood culture growth. Previous diagnostic accuracy studies have shown that patients with equivocal scores encompass cases labeled by clinical adjudicators as viral infection, as well as cases labeled by adjudicators as bacterial infection.^6^^,^^7^ Of note, as a patient recovers from a bacterial infection, their MMBV score resolves from high to low, passing through the equivocal range, reflecting their dynamic immune response.^13^ Therefore, some of the patients with equivocal scores may represent recovering bacterial infections, either because of antibiotic treatment or self-resolving. Other patients with equivocal scores may represent patients with undifferentiated immune responses. A deeper understanding of this needs to be addressed by further studies. Regarding clinical decision making, equivocal results are valid (ie, not a test failure) but nonactionable test results.

In a real-world evaluation of MMBV implementation in an urgent care network, 79% alignment was observed between viral MMBV results and not prescribing antibiotics for adults presenting with suspected infection.^9^ Notably, a lower alignment of 63.1% was observed for cases where the physician was considering prescribing antibiotics pre-MMBV and then received a viral MMBV result, which is more similar to the situation here, where the physician has ordered a blood culture in parallel to MMBV. Indeed, although we observed initially lower levels, alignment significantly increased from 19.3% in period 1 to 38.6% in period 2. We suspect that the lower alignment attained in this high-acuity ED in period 2, compared with the national urgent care network, may be due to the complexity of the patient population. Furthermore, we hypothesize that the significant increase in alignment between MMBV and the physician’s antibiotic prescription decision over time for viral MMBV results reflects the physician’s increasing familiarity with the test. Similarly, hospital admission rates for viral cases declined, which may indicate that physicians felt more comfortable managing these patients as outpatients when supported by a viral MMBV score. Notably, these changes in patient management occurred without a significant increase in ED-return visits, supporting that the reduction in antibiotic prescribing and admission did not compromise patient safety. Future qualitative studies are warranted to assess the barriers and drivers of test adoption and impact on practice.

Previously, MMBV availability has been associated with higher antibiotic prescription and fewer adverse outcomes for patients with bacterial MMBV results, supporting that MMBV aids in identifying missed bacterial infections.^9^^,^^10^ Here, there was a significant increase in prescriptions for cases with MMBV bacterial results in time period 2, but the reduction in ED-return visits was not significant. We suspect that the high baseline prescription rate (93.0%) underlies this finding, which is because of the high acuity of the population. Importantly, the high sensitivity of MMBV ensures that it can also be implemented in such a high-acuity setting without compromising patient safety.

The cohorts in the 2 time periods had highly comparable patient characteristics, supporting that MMBV implementation contributes to the observed practice changes. There was an increase in ordering of rapid viral PCR tests in period 2, but without a significant difference in the rates of viral pathogen detection. Therefore, it seems unlikely that rapid viral PCR testing explains the observed reduction in antibiotic prescribing. This premise aligns with cumulating studies establishing the limited impact of viral PCRs on antibiotic stewardship.^14^^,^^15^

In conclusion, the test’s high sensitivity, shown here against clinically relevant blood cultures, supports MMBV’s use in reducing diagnostic uncertainty and likely contributed to its growing clinical impact over time.

## Author Contributions

A.L., L.K., B.L., E.E., T.G., and S.M. contributed to design the trial, execution, analysis of the data, and draft and review the manuscript. A.L., M.G., H.B., and M.C. supervised the data collection and managed the data, including quality control. S.M. takes responsibility for the paper as a whole.

## Funding and Support

This study is an investigator-initiated study conducted by Maimonides Medical Center, with data extraction funded by MeMed.

## Conflicts of Interest

Kellerman, Lebedenko, Eden, and Gottlieb are employees of MeMed, some of whom have options. Dr Motov is participating in a randomized control trial of MMBV performance sponsored by MeMed. LoVerde, Ghitan, Bodenstein, and Christian have no conflict of interest to disclose.
