# Supplementary material for: Real-World Study Assessing Sensitivity and Clinical Utility of a Host-Protein Test in Adult Emergency Department Patients With Blood Culture Ordered
Source: J Am Coll Emerg Physicians Open. 2025 Sep 30;6(6):100255. doi: 10.1016/j.acepjo.2025.100255 (PMC12514503; doi:10.1016/j.acepjo.2025.100255)
Supplement: Supplementary Material [file mmc1.docx]

**Real world study assessing sensitivity and clinical utility of a host-protein test in adult ED patients with blood culture ordered**

# Supplementary Material:

Statistical framework for performance parameters:

Sensitivity, specificity, negative predictive value (NPV), and positive predictive value (PPV) were calculated based on two predefined score thresholds with a bacterial infection considered ‘positive’. Cases with MMBV scores >65 were classified as bacterial infections (including co-infections), while those with scores <35 were classified as viral infections (or other non-bacterial etiologies). Cases with scores between 35 and 65 were classified as equivocal, removed from these calculations and are given as a rate.

Supplementary Table 1 Diagnostic accuracy of MMBV in comparison to clinically relevant blood cultures

|  | | MMBV Results | |
| --- | --- | --- | --- |
|  |  | Bacterial | Viral |
| Clinically Relevant Blood Cultures | Positive | TP= 53 (96.4%) | FN = 2 (3.6%) |
|  | Negative (+contamination) | FP = 408 (75.0%) | TN = 136 (25.0%) |

Sensitivity (95% CI): 96.4% (87.0 - 99.7)
Specificity (95% CI): 25.0% (21.5 - 28.8)
Positive Predictive Value (95% CI): 11.5% (8.9 - 14.7)
Negative Predictive Value (95% CI): 98.6% (94.5 - 99.9)

LR(+): 1.285 (1.197-1.379)

LR(-): 0.145 (0.037-0571)
Equivocal: (82/681) 12.0%

A bacterial infection is considered ‘positive’.

Supplementary Table 2: Clinically relevant culture cases with viral and equivocal MMBV results.


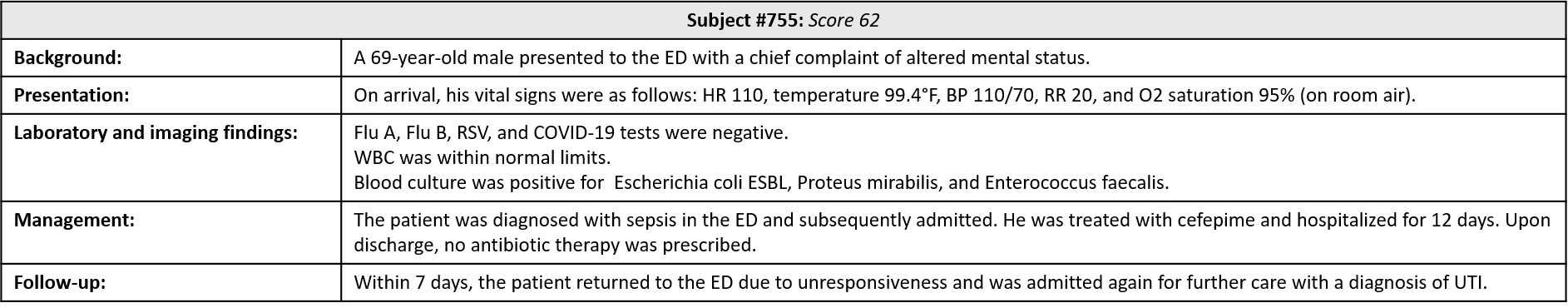

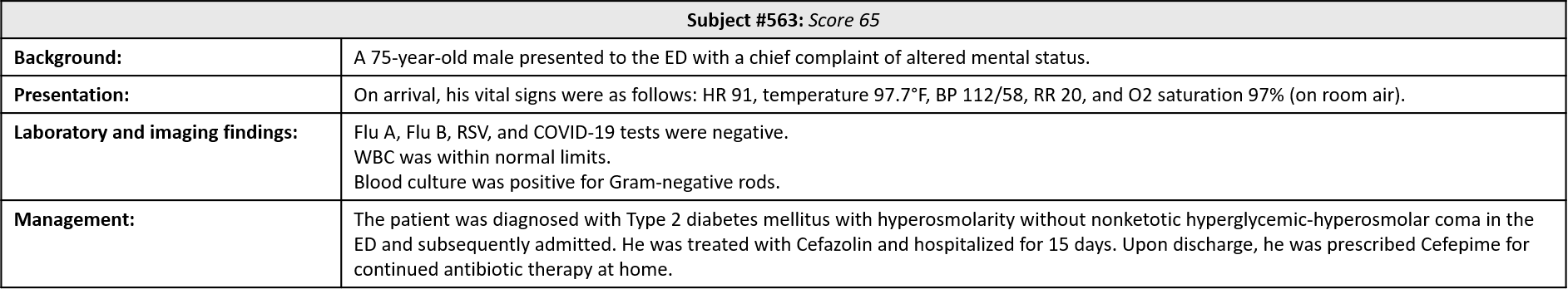

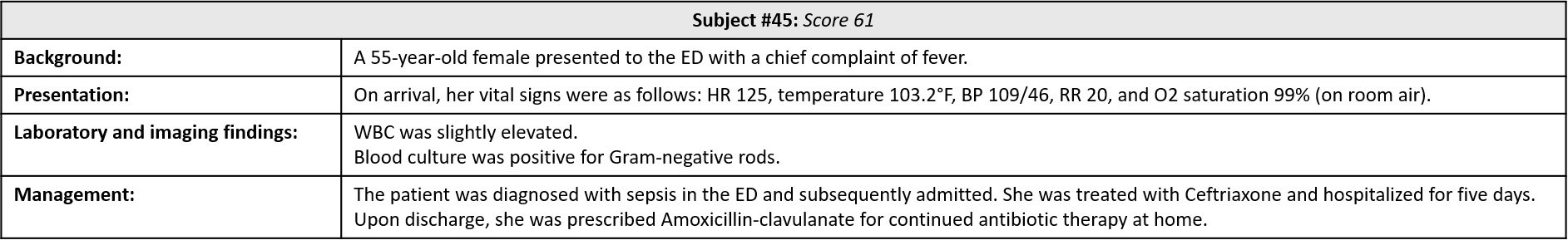

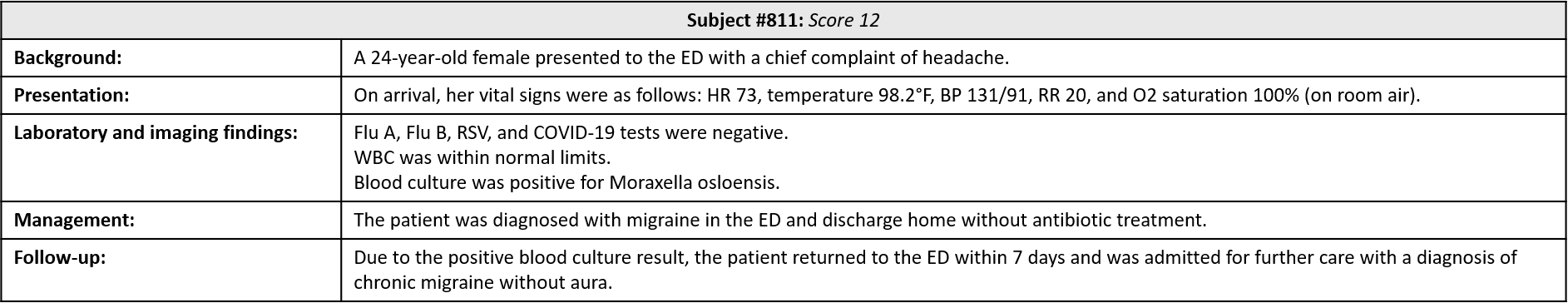

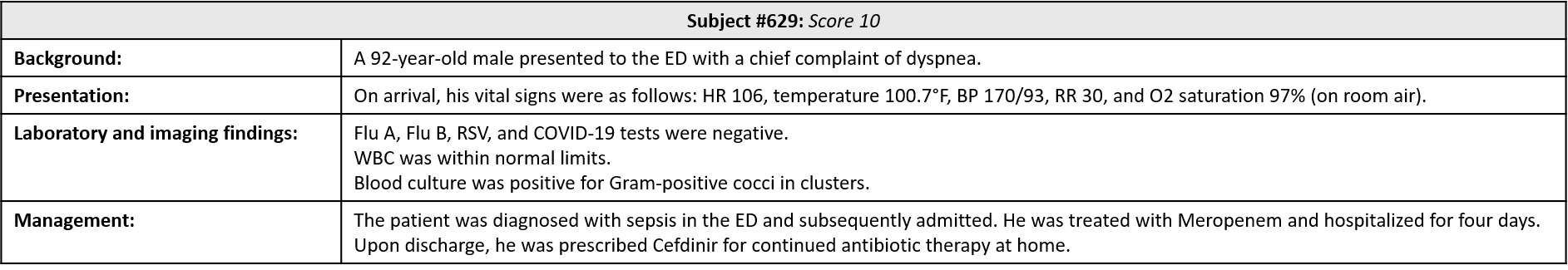


Supplementary Table 3: Patient characteristics across two-time matching time periods stratified according to MMBV results.

|  |  | **Bacterial** | | | **Equivocal** | | | **Viral** | | | |
| --- | --- | --- | --- | --- | --- | --- | --- | --- | --- | --- | --- |
|  |  | Apr 22 - Mar 23 (N=227) | Apr 23 - Mar 24 (N=206) | p-value | Apr 22 - Mar 23 (N=38) | Apr 23 - Mar 24 (N=41) | p-value | Apr 22 - Mar 23 (N=88) | Apr 23 - Mar 24 (N=44) | p-value |  |
| Demographics | Age, median (IQR) | 71.0 (57.0, 82.5) | 74.0 (58.0, 84.8) | 0.394 | 75.0 (63.2, 85.5) | 73.0 (53.0, 85.0) | 0.438 | 68.0 (46.8, 81.0) | 72.0 (45.5, 80.0) | 0.898 |  |
|  | Sex (male), n (%) | 117 (51.5%) | 121 (58.7%) | 0.133 | 19 (50.0%) | 13 (31.7%) | 0.100 | 45 (51.1%) | 26 (59.1%) | 0.389 |  |
| Current illness | Temperature (C), mean (SD) | 37.7 (1.1) | 37.5 (1.1) | 0.078 | 37.2 (0.8) | 37.1 (0.9) | 0.806 | 37.6 (1.2) | 37.7 (1.0) | 0.697 |  |
|  | ED LOS (hours), mean (SD) | 8.6 (6.5) | 8.6 (5.8) | 0.896 | 9.4 (7.9) | 8.9 (7.0) | 0.752 | 8.0 (5.1) | 7.7 (5.1) | 0.758 |  |
| Chief complaint | Fever, n (%) | 64 (28.2%) | 47 (22.8%) | 0.201 | 7 (18.4%) | 5 (12.2%) | 0.444 | 19 (21.6%) | 12 (27.9%) | 0.426 |  |
|  | dyspnea, n (%) | 37 (16.3%) | 39 (18.9%) | 0.473 | 12 (31.6%) | 11 (26.8%) | 0.645 | 20 (22.7%) | 6 (14.0%) | 0.239 |  |
|  | altered mental status, n (%) | 22 (9.7%) | 24 (11.7%) | 0.509 | 5 (13.2%) | 7 (17.1%) | 0.630 | 8 (9.1%) | 2 (4.7%) | 0.371 |  |
|  | weakness/fatigue/lethargy, n (%) | 12 (5.3%) | 17 (8.3%) | 0.218 | 1 (2.6%) | 5 (12.2%) | 0.111 | 4 (4.5%) | 3 (7.0%) | 0.563 |  |
|  | cough, n (%) | 8 (3.5%) | 7 (3.4%) | 0.943 | 2 (5.3%) | 1 (2.4%) | 0.514 | 11 (12.5%) | 3 (7.0%) | 0.338 |  |
|  | abdominal pain, n (%) | 13 (5.7%) | 11 (5.3%) | 0.861 | 1 (2.6%) | 1 (2.4%) | 0.957 | 4 (4.5%) | 1 (2.3%) | 0.535 |  |
| Tests | CBC ordered, n (%) | 224 (98.7%) | 204 (99.0%) | 0.733 | 38 (100.0%) | 40 (97.6%) | 0.336 | 87 (98.9%) | 44 (100.0%) | 0.480 |  |
|  | WBC, median (IQR) | 11.4 (8.4, 15.5) | 12.0 (8.6, 15.7) | 0.544 | 9.6 (6.8, 12.7) | 7.6 (5.9, 11.9) | 0.261 | 6.9 (5.2, 9.4) | 7.6 (5.7, 9.2) | 0.388 |  |
|  | Procalcitonin, median (IQR) | 1.4 (0.6, 4.2) | 1.2 (0.6, 4.7) | 0.994 | 0.4 (0.3, 0.6) | 0.6 (0.4, 1.1) | 0.173 | 0.5 (0.4, 2.2) | 1.1 (1.0, 1.2) | 0.334 |  |
|  | CRP ordered, n (%) | 19 (8.4%) | 25 (12.1%) | 0.196 | 7 (18.4%) | 4 (9.8%) | 0.269 | 19 (21.6%) | 5 (11.4%) | 0.153 |  |
|  | CRP (mg/dL), median (IQR) | 11.4 (3.6, 15.6) | 10.0 (6.8, 15.6) | 0.492 | 10.7 (2.5, 11.4) | 3.0 (1.0, 5.9) | 0.230 | 1.3 (0.7, 3.1) | 1.3 (0.4, 2.0) | 0.581 |  |
|  | Broader viral PCR test ordered, n (%) | 25 (11.0%) | 22 (10.7%) | 0.911 | 3 (7.9%) | 6 (14.6%) | 0.349 | 7 (8.0%) | 1 (2.3%) | 0.199 |  |
|  | Rapid viral panel test ordered, n (%) | 115 (50.7%) | 153 (74.3%) | <0.001 | 18 (47.4%) | 23 (56.1%) | 0.441 | 43 (48.9%) | 35 (79.5%) | <0.001 |  |
|  | Influenza A, n (%) | 1 (0.9%) | 4 (2.6%) | 0.297 | 0 (0.0%) | 0 (0.0%) | NA | 4 (9.3%) | 6 (17.1%) | 0.306 |  |
|  | Influenza B, n (%) | 0 (0.0%) | 1 (0.7%) | 0.386 | 0 (0.0%) | 0 (0.0%) | NA | 1 (2.3%) | 0 (0.0%) | 0.367 |  |
|  | Covid-19, n (%) | 7 (6.1%) | 7 (4.6%) | 0.583 | 1 (5.6%) | 3 (13.0%) | 0.428 | 13 (31.0%) | 5 (14.3%) | 0.087 |  |
|  | RSV, n (%) | 1 (0.9%) | 2 (1.3%) | 0.737 | 1 (5.6%) | 0 (0.0%) | 0.258 | 1 (2.3%) | 0 (0.0%) | 0.367 |  |
| Microbiological testing | Blood culture ordered, n (%) | 227 (100.0%) | 206 (100.0%) | NA | 38 (100.0%) | 41 (100.0%) | NA | 88 (100.0%) | 44 (100.0%) | NA |  |
|  | Blood culture results: Contaminant, n (%) | 10 (4.4%) | 11 (5.3%) | 0.652 | 0 (0.0%) | 1 (2.4%) | 0.336 | 5 (5.7%) | 1 (2.3%) | 0.377 |  |
|  | Blood culture results: Negative, n (%) | 187 (82.4%) | 175 (85.0%) | 0.471 | 38 (100.0%) | 38 (92.7%) | 0.091 | 83 (94.3%) | 41 (93.2%) | 0.797 |  |
|  | Blood culture results: Positive, n (%) | 30 (13.2%) | 20 (9.7%) | 0.255 | 0 (0.0%) | 2 (4.9%) | 0.171 | 0 (0.0%) | 2 (4.5%) | 0.045 |  |
| MMBV | MMBV Score, median (IQR) | 97.0 (89.5, 100.0) | 97.0 (89.0, 100.0) | 0.950 | 49.0 (42.0, 58.0) | 49.0 (43.0, 56.0) | 0.965 | 5.0 (2.0, 18.0) | 9.5 (2.0, 13.2) | 0.931 |  |
|  | MMBV Result: Bacterial, n (%) | 227 (100.0%) | 206 (100.0%) | NA | 0 (0.0%) | 0 (0.0%) | NA | 0 (0.0%) | 0 (0.0%) | NA |  |
|  | MMBV Result: Equivocal, n (%) | 0 (0.0%) | 0 (0.0%) | NA | 38 (100.0%) | 41 (100.0%) | NA | 0 (0.0%) | 0 (0.0%) | NA |  |
|  | MMBV Result: Viral, n (%) | 0 (0.0%) | 0 (0.0%) | NA | 0 (0.0%) | 0 (0.0%) | NA | 88 (100.0%) | 44 (100.0%) | NA |  |
|  | Alignment, n (%) | 211 (93.0%) | 202 (98.1%) | 0.012 | NaN | NaN | NaN | 17 (19.3%) | 17 (38.6%) | 0.017 |  |
| Hospitalization | Hospital admission, n (%) | 204 (89.9%) | 190 (92.2%) | 0.391 | 30 (78.9%) | 33 (80.5%) | 0.866 | 68 (77.3%) | 27 (61.4%) | 0.056 |  |
|  | Hospitalization duration, mean (SD) | 8.6 (10.7) | 10.1 (11.1) | 0.192 | 8.2 (8.1) | 8.7 (5.6) | 0.777 | 6.6 (6.4) | 6.6 (6.4) | 0.974 |  |
| Antibiotics | Antibiotics prescribed in ED, n (%) | 211 (93.0%) | 202 (98.1%) | 0.012 | 33 (86.8%) | 33 (80.5%) | 0.450 | 71 (80.7%) | 27 (61.4%) | 0.017 |  |
| Discharge Diagnosis | Infectious Diseases, n (%) | 91 (40.1%) | 66 (32.0%) | 0.082 | 6 (15.8%) | 12 (29.3%) | 0.156 | 26 (29.5%) | 12 (27.3%) | 0.787 |  |
|  | Respiratory Disorders, n (%) | 16 (7.0%) | 18 (8.7%) | 0.514 | 8 (21.1%) | 6 (14.6%) | 0.458 | 13 (14.8%) | 4 (9.1%) | 0.360 |  |
|  | Cardiovascular Disorders, n (%) | 15 (6.6%) | 18 (8.7%) | 0.405 | 5 (13.2%) | 3 (7.3%) | 0.393 | 9 (10.2%) | 4 (9.1%) | 0.837 |  |
|  | Hematological and Immunological Disorders, n (%) | 17 (7.5%) | 25 (12.1%) | 0.103 | 3 (7.9%) | 2 (4.9%) | 0.585 | 5 (5.7%) | 2 (4.5%) | 0.784 |  |
|  | Endocrine and Metabolic Disorders, n (%) | 16 (7.0%) | 17 (8.3%) | 0.638 | 3 (7.9%) | 4 (9.8%) | 0.773 | 4 (4.5%) | 1 (2.3%) | 0.521 |  |
| Return Visits | Return visit within 7 days, n (%) | 22 (9.7%) | 13 (6.3%) | 0.198 | 3 (7.9%) | 6 (14.6%) | 0.349 | 4 (4.5%) | 4 (9.1%) | 0.304 |  |

STARD checklist

|  | **Section & Topic** | **No** | **Item** | **Reported on page #** |
| --- | --- | --- | --- | --- |
|  |  |  |  |  |
|  | **TITLE OR ABSTRACT** |  |  |  |
|  |  | **1** | Identification as a study of diagnostic accuracy using at least one measure of accuracy  (such as sensitivity, specificity, predictive values, or AUC) | 1 |
|  | **ABSTRACT** |  |  |  |
|  |  | **2** | Structured summary of study design, methods, results, and conclusions  (for specific guidance, see STARD for Abstracts) | 1 |
|  | **INTRODUCTION** |  |  |  |
|  |  | **3** | Scientific and clinical background, including the intended use and clinical role of the index test | 2 |
|  |  | **4** | Study objectives and hypotheses | 2 |
|  | **METHODS** |  |  |  |
|  | *Study design* | **5** | Whether data collection was planned before the index test and reference standard  were performed (prospective study) or after (retrospective study) | 3 |
|  | *Participants* | **6** | Eligibility criteria | 3 |
|  |  | **7** | On what basis potentially eligible participants were identified  (such as symptoms, results from previous tests, inclusion in registry) | 3 |
|  |  | **8** | Where and when potentially eligible participants were identified (setting, location and dates) | 3 |
|  |  | **9** | Whether participants formed a consecutive, random or convenience series | 3 |
|  | *Test methods* | **10a** | Index test, in sufficient detail to allow replication | 3 |
|  |  | **10b** | Reference standard, in sufficient detail to allow replication | 3 |
|  |  | **11** | Rationale for choosing the reference standard (if alternatives exist) | 2 |
|  |  | **12a** | Definition of and rationale for test positivity cut-offs or result categories  of the index test, distinguishing pre-specified from exploratory | 3, supplementary |
|  |  | **12b** | Definition of and rationale for test positivity cut-offs or result categories  of the reference standard, distinguishing pre-specified from exploratory | 3, supplementary |
|  |  | **13a** | Whether clinical information and reference standard results were available  to the performers/readers of the index test | 3, 4 |
|  |  | **13b** | Whether clinical information and index test results were available  to the assessors of the reference standard | 3, 4 |
|  | *Analysis* | **14** | Methods for estimating or comparing measures of diagnostic accuracy | 4, supplementary |
|  |  | **15** | How indeterminate index test or reference standard results were handled | 4, supplementary |
|  |  | **16** | How missing data on the index test and reference standard were handled | 3 |
|  |  | **17** | Any analyses of variability in diagnostic accuracy, distinguishing pre-specified from exploratory | supplementary |
|  |  | **18** | Intended sample size and how it was determined | n/a |
|  | **RESULTS** |  |  |  |
|  | *Participants* | **19** | Flow of participants, using a diagram | 12 |
|  |  | **20** | Baseline demographic and clinical characteristics of participants | 4, 5, 11 |
|  |  | **21a** | Distribution of severity of disease in those with the target condition | Table 1 |
|  |  | **21b** | Distribution of alternative diagnoses in those without the target condition | Table 1 |
|  |  | **22** | Time interval and any clinical interventions between index test and reference standard | n/a |
|  | *Test results* | **23** | Cross tabulation of the index test results (or their distribution)  by the results of the reference standard | 5, supplementary |
|  |  | **24** | Estimates of diagnostic accuracy and their precision (such as 95% confidence intervals) | 5, supplementary |
|  |  | **25** | Any adverse events from performing the index test or the reference standard | 3 |
|  | **DISCUSSION** |  |  |  |
|  |  | **26** | Study limitations, including sources of potential bias, statistical uncertainty, and generalisability | 6 |
|  |  | **27** | Implications for practice, including the intended use and clinical role of the index test | 6, 7 |
|  | **OTHER INFORMATION** |  |  |  |
|  |  | **28** | Registration number and name of registry | 3 |
|  |  | **29** | Where the full study protocol can be accessed | n/a |
|  |  | **30** | Sources of funding and other support; role of funders | DECLARATION OF INTERESTS |
|  |  |  |  |  |
